# Efficacy and safety of DFN-11 (sumatriptan injection, 3 mg) in adults with episodic migraine: an 8-week open-label extension study

**DOI:** 10.1186/s10194-018-0882-y

**Published:** 2018-08-15

**Authors:** Stephen Landy, Sagar Munjal, Elimor Brand-Schieber, Alan M. Rapoport

**Affiliations:** 1 Baptist Medical Group Headache Clinic, University of Tennessee Medical School, 6029 Walnut Grove, Suite 210, Memphis, TN 38120 USA; 2 Promius Pharma, LLC, a subsidiary of Dr. Reddy’s Laboratories, 107 College Road East, Princeton, NJ 08540 USA; 3 The David Geffen School of Medicine at UCLA, 4255 Jefferson Avenue, Suite 27, Woodside, CA 94062 USA

**Keywords:** Low-dose sumatriptan, Multi-attack acute subcutaneous sumatriptan, Consistency of effect

## Abstract

**Background:**

DFN-11, a 3 mg sumatriptan subcutaneous (SC) autoinjector for acute treatment of migraine, has not been assessed previously in multiple attacks. The objective of this study was to evaluate the efficacy, tolerability, and safety of DFN-11 in the acute treatment of multiple migraine attacks.

**Methods:**

This was an 8-week open-label extension of multicenter, randomized, double-blind, placebo-controlled US study. Subjects averaging 2 to 6 episodic migraine attacks per month were randomized to DFN-11 or placebo to treat a single attack of moderate-to-severe intensity and then entered the extension study to assess the efficacy, tolerability, and safety of DFN-11 in multiple attacks of any pain intensity.

**Results:**

Overall, 234 subjects enrolled in the open-label period, and 29 (12.4%) discontinued early. A total of 848 migraine episodes were treated with 1042 doses of open-label DFN-11 and subjects treated a mean (SD) of 3.9 (2.3) attacks. At 2 h postdose in attacks 1 (*N* = 216), 2 (*N* = 186), 3 (*N* = 142) and 4 (*N* = 110), respectively, pain freedom rates were 57.6%, 64.6%, 61.6%, and 66.3%; pain relief rates were 83.4%, 88.4%, 84.1%, and 81.7%; most bothersome symptom (MBS)-free rates were 69.0%, 76.5%, 77.7%, and 74.7%; nausea-free rates were 78.1%, 84.6%, 86.5%, and 85.7%; photophobia-free rates were 75.3%, 76.4%, 72.3%, and 77.5%; and phonophobia-free rates were 75.2%, 77.5%, 73.6%, and 76.0%. Overall, 40.6% (89/219) of subjects reported treatment-emergent adverse events (TEAE), the most common of which were associated with the injection site: swelling (12.8%), pain (11.4%), irritation (6.4%), and bruising (6.4%). Most subjects (65.2%, 58/89) had mild TEAEs; severe TEAEs were reported by 1 subject (treatment-related jaw tightness). Five subjects (2.1%) discontinued due to adverse events, which included mild throat tightness (*n* = 2), moderate hernia pain (*n* = 1), moderate hypersensitivity (*n* = 1), and 1 subject with mild nausea and moderate injection site swelling. There were no serious TEAEs and no new or unexpected safety findings.

**Conclusion:**

DFN-11 was effective, tolerable, and safe in the acute treatment of 4 migraine attacks over 8 weeks, with consistent responses on pain and associated symptoms. Most TEAEs were mild, with a very low incidence of triptan-related TEAEs. DFN-11 is potentially an effective and safe alternative for the acute treatment of migraine.

**Trial registration:**

ClinicalTrials.gov, NCT02569853. Registered 07 October 2015.

## Background

Migraine is a painful, disabling, and, for most patients, lifelong disease [1, 2]. Although migraineurs rate consistent relief with few side effects among the most desirable attributes of an acute migraine medication [3–6], and clinical trial guidelines recommend assessment of the consistency of response to acute medications in multiple-attack studies [7], the effectiveness of acute medications across multiple attacks is not frequently evaluated in clinical trials. Yet the utility of acute treatments depends, in part on their ability to be effective, tolerable, and safe over the long-term [8]; inconsistent relief is an important reason for dissatisfaction with acute therapy [3]. Moreover, confidence that an intervention will reliably relieve migraine pain and associated symptoms is a predictor of adherence to acute therapy [9]. Single-attack studies are not designed to evaluate inter-attack variability of treatment outcomes [10].

DFN-11 (Zembrace® SymTouch®, Promius Pharma, Princeton, NJ) is a low-dose (3 mg) SC sumatriptan injection, supplied as a single-dose, ready-to-use, disposable autoinjector. Compared with the 6-mg SC dose of sumatriptan (Imitrex®, GlaxoSmithKline, Research Triangle Park, NC), DFN-11 has less sumatriptan per 0.5-mL dose (3 mg vs 6 mg) [11, 12]. Other research has shown that DFN-11 provides relief of migraine pain and associated symptoms similar to a 6-mg SC dose of sumatriptan, with fewer triptan sensations and no reports of chest pain, in adults with rapidly-escalating migraine attacks [13]. Subsequent work in episodic migraine found that DFN-11 was significantly more effective than placebo on pain-free and pain relief outcomes from 30 min through 2 h postdose and confirmed the low incidence of TEAEs and triptan sensations [14]. The objective of this study was to evaluate the efficacy, tolerability, and safety of DFN-11 in the acute treatment of multiple migraine attacks in adults with episodic migraine.

## Methods

### Ethics

This was a randomized, double-blind, placebo-controlled study with an open-label extension to evaluate the efficacy, tolerability, and safety of DFN-11 in adults with episodic migraine at 16 US study centers. The data from the double-blind portion of the study have been presented elsewhere [14]. The protocol was approved by the institutional review boards at each study site, and the study conduct complied with good clinical practice and the ethical principles in the Declaration of Helsinki. Prior to screening, investigators explained the nature of the study and obtained informed consent from subjects. The study is registered at ClinicalTrials.gov (https://clinicaltrials.gov/; Identifier NCT02569853).

### Subjects

Subjects included adult males and females (18–65 years of age) with a history of episodic migraine with or without aura (defined by the Second Edition of International Classification of Headache Disorders (ICHD-2) [15]). They had to have 2 to 6 migraine attacks per month for at least the previous 12 months, with no more than 14 headache days per month and a minimum of 48 h of headache-free time between attacks. Subjects had to meet all inclusion and exclusion criteria to be included in the study.

### Treatments

DFN-11 (equivalent to 3 mg sumatriptan base in 0.5 mL sterile solution) was provided as an SC injection in a 29-gauge needle-based autoinjector.

### Study procedures

This study included site visits for screening, baseline/randomization, end double-blind/begin open-label, week 4 ± 3 days, and week 8 ±3 days/early termination.

During screening, subjects provided informed consent and staff verified inclusion and exclusion criteria. Subjects were given an electronic diary (eDiary) and instructions on how to complete it, medical and migraine histories were taken, and a physical examination was performed.

At baseline, inclusion and exclusion criteria were re-verified, and medical and treatment histories and physical examinations (including laboratory and vital sign measurements) were repeated. Subjects were randomized (1:1) to receive DFN-11 or placebo via SC autoinjector in a double-blinded fashion to treat 1 migraine attack. Study centers used an Interactive Web Response System to assign drug kits (ie, labeled cartons containing 2 individually labeled autoinjectors) at scheduled and unscheduled visits as needed.

At the conclusion of the double-blind treatment period, subjects were re-examined, and vital signs measurements were repeated. Study staff assessed eligibility for continuing into an open-label period, and eligible subjects entered an 8-week open-label period. During this period, subjects received DFN-11 for 8 weeks and were instructed to treat multiple attacks within 1 h of migraine pain onset at any level of pain intensity. If subjects did not experience sufficient relief 2 h after taking the first dose of study medication, they were allowed a second dose of study medication or rescue medication for the same attack. No more than 2 doses of study medication could be taken in any 24-h period. Rescue medications could include prescription and nonprescription drugs (eg, NSAIDs, other acute migraine medications, vitamins, herbal/dietary supplements).

Adverse events (AEs) were monitored from the time subjects gave informed consent; physical examinations, vital sign measurements, ECGs, and laboratory assessments were performed at designated site visits.

### Assessments

In the open-label period, efficacy was assessed for each of the first 4 reported migraine attacks. Efficacy endpoints included the percentage of subjects who had pain-freedom, pain relief, and absence of their most bothersome symptom (MBS) at 10, 15, 20, 30, 60, 90, and 120 min, and the percentage of subjects who were free from nausea, photophobia, and phonophobia at 2 h postdose. The percentage of subjects with sustained pain freedom from 2 to 24 h postdose was also assessed.

Pain freedom was defined as a reduction in migraine pain from a predose rating of moderate (Grade 2) or severe (Grade 3) pain to none (Grade 0). Pain relief was defined as a reduction in migraine pain from predose rating of severe (Grade 3) or moderate (Grade 2) to mild pain (Grade 1) or none (Grade 0), or from mild pain (Grade 1) to none (Grade 0). Absence of MBS was defined as absence of the symptom chosen as most bothersome from among nausea, photophobia, or phonophobia at predose. Sustained pain freedom was defined as pain-free at 2 h postdose with no use of rescue medication or additional study medication and no recurrence of headache pain within 2 to 24 h postdose.

Safety and tolerability were assessed throughout the open-label period. Tolerability included the percentage of subjects with treatment-emergent AEs (TEAEs). Safety endpoints included the percentage of subjects with serious AEs (SAEs), as well as those with changes in vital signs or ECGs. Safety parameters included concomitant medication review; physical examinations; pregnancy tests in females; measurement of vital signs (sitting systolic and diastolic blood pressure, pulse rate, and body temperature); clinical laboratory examination (hematology, chemistry, and urinalysis); urine drug screen; and 12-lead ECG.

### Statistics

All data processing, summarization, and analyses were performed using SAS® software, Version 9.2. Adverse events were classified using the Medical Dictionary for Regulatory Activities (MedDRA) dictionary, Version 18.0. Concomitant medications were coded using the World Health Organization Drug Dictionary Enhanced (WHODDE), version Mar2015 further coded against Anatomic Therapeutic Chemical (ATC) classification.

Subjects randomized in the double-blind period comprised the population for subject disposition and baseline summaries. The open-label efficacy analyses included all open-label subjects who received at least 1 dose of active study medication and recorded at least 1 postdose efficacy data point in the open-label period. The open-label safety analysis included all open-label subjects who received at least 1 dose of study medication.

Unless noted otherwise, a last observation carried forward (LOCF) imputation method was applied to pain intensity and the presence of nausea, photophobia, and phonophobia. Baseline data were not carried forward, and only valid data from postbaseline assessments collected before the 2-h postdose time point were carried forward to impute the next missing assessment up to the 2-h postdose time point. Time points beyond 2 h postdose were not carried forward.

The analysis of safety was based on data from all randomized subjects who received at least 1 dose of study medication. The efficacy analyses were based on data captured in the eDiary for migraine attacks treated. Postdosing assessments were collected in real-time. Change from baseline was defined as the postbaseline value minus the predose value, and calculations were based on nonmissing data. Baseline for safety assessments was defined as the last assessment before receiving the first dose of study medication in the double-blind period.

## Results

### Disposition

A total of 16 US study sites participated and randomized subjects into the study. The duration of the study, from the first subject’s enrollment until the last subject’s completion, was 618 days (21 September 2015 through 30 May 2017).

**Fig. 1:**
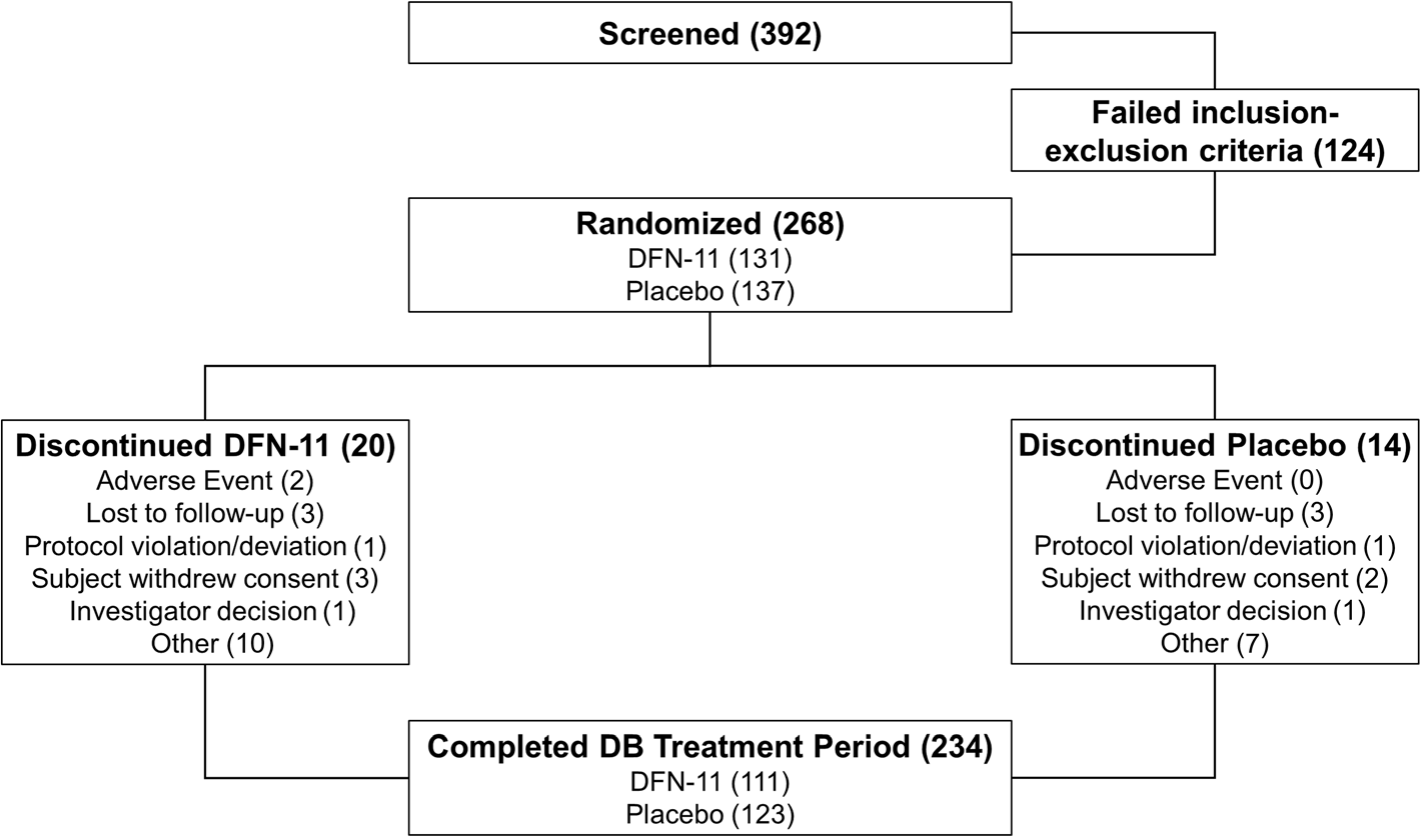
Disposition of subjects

As shown in Fig. 1, 392 subjects were screened, 268 (68.4%) were randomized, 234 (87.3% of those randomized) completed the double-blind treatment period and enrolled in the open-label extension. A total of 205 (87.6% of those who enrolled) completed the open-label extension (Table 1).

**Table 1:** Subject disposition

Screened					392	
Double-blind Treatment Period	DFN-11		Placebo		Total	
Randomized	131		137		268	
Completed	111		123		234	
Discontinued	20		14		34	
Open-label Treatment Period (All active DFN-11)	Treatment Received During DB, Prior to OL Period				Overall	
	DFN-11		Placebo			
Enrolled	111^a^		123^a^		234	
Completed	96^a^		109^a^		205	
Discontinued	15^a^		14^a^		29	
Reasons for discontinuation	Discont. During DB	Discont. During OLE^a^	Discont. During DB	Discont. During OLE^a^	OLE Total	DB + OLE
Adverse event	2	3	0	2	5	7
Lost to follow-up	3	8	3	1	9	15
Protocol violation	1	1	1	3	4	6
Withdrew consent	3	2	2	5	7	12
Investigator decision	1	0	1	2	2	4
Other	10	1	7	1	2	19
Total discontinued	20	15	14	14	29	63

*DB* double-blind treatment period, *OLE* open-label extension period

^a^Treatment group reflects OLE subjects’ assignment during the DB period

Of the 234 subjects who entered the open-label extension, 216 (92.3%) treated at least 1 attack, 186 (79.5%) treated at least 2 attacks, 142 (60.7%) treated at least 3 attacks, and 110 (47.0%) treated 4 attacks or more. A total of 29 subjects (12.4%) discontinued: 9 (3.8%) were lost to follow-up, 7 (3.0%) withdrew consent, and 5 (2.1%) discontinued due to AEs.

### Demographics

Most subjects were female (85.4%) and white (75.7%), with a mean (SD) age of 41.0 (12.4) years. Mean (SD) weight was 84.4 (23.7) kg and mean (SD) BMI was 30.6 (8.6) kg/m^2^.

### Exposure

Over the course of the 8-week open-label extension, subjects used 1042 doses of DFN-11 to treat 848 migraine attacks, and they treated a mean (SD) of 3.9 (2.3) attacks per subject. In attacks 1, 2, 3, and 4, respectively, a second dose of DFN-11 (allowed after the completion of the 2-h efficacy assessments) was used by 19.9% (43/216), 21.0% (39/186), 15.5% (22/142), and 26.4% (29/110) of subjects.

### Efficacy

**Fig. 2:**
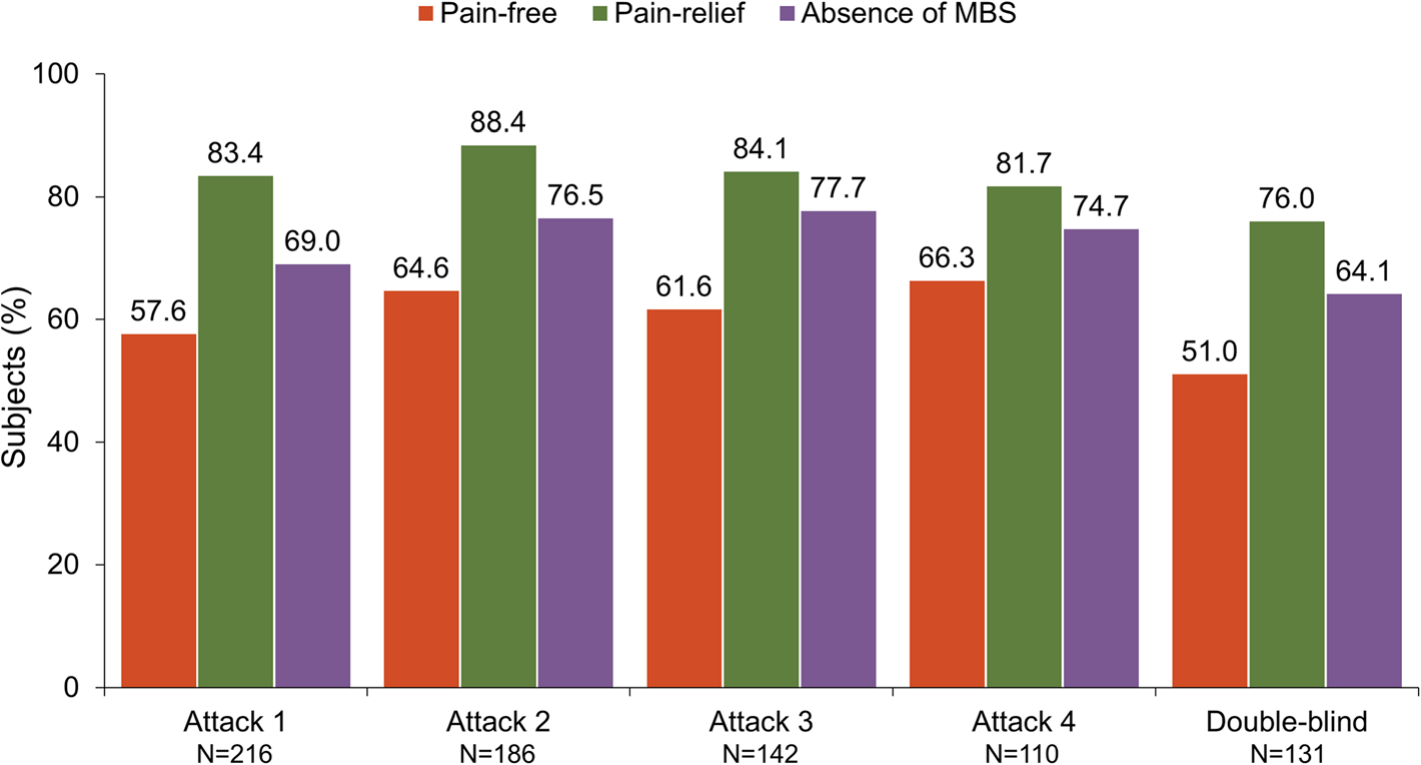
Pain freedom, pain relief, and absence of the most bothersome symptom (MBS) at 2 h after treatment with DFN-11 in the open-label and double-blind study periods

At 2 h after DFN-11 treatment, the percentages of subjects who were pain-free in attacks 1, 2, 3, and 4, respectively, were 57.6%, 64.6%, 61.6%, and 66.3%. The 2-h pain relief response rates were 83.4% for attack 1, 88.4% for attack 2, 84.1% for attack 3, and 81.7% for attack 4. The percentage of subjects with 2-h postdose absence of MBS in attacks 1 to 4, respectively, was 69.0%, 76.5%, 77.7%, and 74.7%. Pain-free, pain relief, and MBS responses to DFN-11 for the 4 individual attacks, as well as for the attack treated with DFN-11 in the double-blind period, are presented in Fig. 2.

**Fig. 3:**
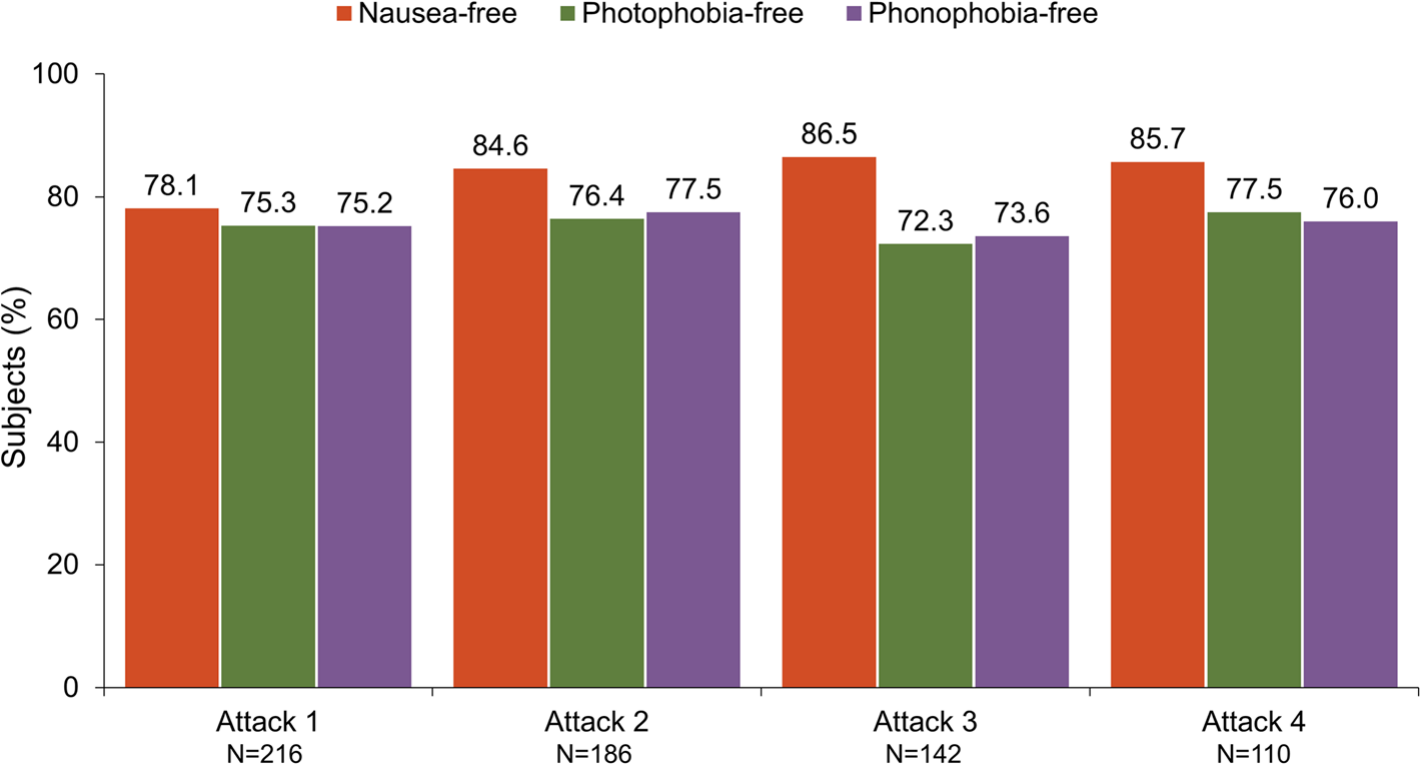
Freedom from nausea, photophobia, and phonophobia at 2 h after treatment with DFN-11

For freedom from the associated symptoms of migraine, Fig. 3 shows that in attacks 1, 2, 3, and 4, respectively, 78.1%, 84.6%, 86.5%, and 85.7% subjects were free of nausea; 75.3%, 76.4%, 72.3%, and 77.5% were free of photophobia; and 75.2%, 77.5%, 73.6%, and 76.0% were free of phonophobia.

Rates of sustained pain freedom from 2 to 24 h postdose in attacks 1, 2, 3, and 4, respectively, were 83.9% (78/93), 76.5% (65/85), 81.3% (52/64), and 77.8% (42/54).

#### Use of a second dose or rescue medication

The percentage of subjects who took a second dose of study medication, rescue medication, or both, in the 2 to 24 h postdose in attacks 1, 2, 3, and 4, respectively, was 19.4% (42/216), 21.0% (39/186), 16.9% (24/142), and 25.5% (28/110).

### Tolerability and safety

During the open-label period, 40.6% (89/219) of subjects reported TEAEs. The most common TEAEs were injection site swelling (12.8%), injection site pain (11.4%), injection site irritation (6.4%), and injection site bruising (6.4%), as shown in Table 2. Most subjects (65.2%, 58/89) reported a maximum TEAE severity of mild; 24.7% (22/89) reported moderate TEAEs. Eight subjects did not have severity assigned. A single subject reported 10 occurrences of severe joint stiffness (described as injection-related jaw tightness) that was considered definitely related to the study medication. The relationship to DFN-11 was considered by the investigator as definite in 26.9% (59/219) of subjects and probable in 6.4% (14/219) of subjects. Five subjects (2.1%) discontinued due to AEs that included mild throat tightness (*n* = 2); moderate hernia pain (*n* = 1); moderate hypersensitivity (*n* = 1); and mild nausea and moderate injection site swelling (*n* = 1).

**Table 2:** Treatment-emergent adverse events occurring in ≥1% of subjects treated with DFN-11

	(*N* = 219)
	n (%)
Subjects with ≥1 TEAE	89 (40.6)
Injection site	
Bruising	14 (6.4)
Erythema	8 (3.7)
Induration	6 (2.7)
Irritation	14 (6.4)
Pain	25 (11.4)
Swelling	28 (12.8)
Nausea	6 (2.7)
Chest discomfort	6 (2.7)
Sinusitis	3 (1.4)
Upper respiratory tract infection	7 (3.2)
Burning sensation	3 (1.4)
Dizziness	4 (1.8)
Paresthesia	3 (1.4)
Somnolence	3 (1.4)

*TEAE* treatment-emergent adverse event

There were no deaths or treatment-emergent SAEs, and no notable shifts in chemistry or hematology parameters, vital signs, or physical examinations; no clinically significant values on these parameters were reported during the study.

### Injection site reactions

The most common TEAEs overall were associated with the injection site: swelling (12.8%, 28/219); pain (11.4%, 25/219); and irritation and bruising (both 6.4%, 14/219 each). At least 1 injection site reaction was reported by 27.4% (60/219) of subjects overall, and rates were 19.9% (43/216) in attack 1, 13.4% (25/186) in attack 2, 10.6% (15/142) in attack 3, and 14.5% (16/110) in attack 4.

### Triptan-related adverse events

A total of 12.3% (27/219) of subjects had at least 1 triptan-related AE, with 10.6% (23/216), 9.1% (17/186), 7.7% (11/142), and 7.3% (8/110) of subjects experiencing them in attacks 1, 2, 3, and 4, respectively. Chest discomfort was reported 32 times by a total of 6 subjects (2.7%); 5 subjects had 30 mild events, and the sixth had 1 mild and 1 moderate event. These events were described variously by the study investigators as chest tightness, noncardiac; chest tightness after injection, noncardiac; sensation of chest heaviness after injection, noncardiac; and pressure sensation, chest, noncardiac.

## Discussion

This open-label extension study was conducted to evaluate the efficacy, tolerability, and safety of DFN-11 in the acute treatment of multiple migraine attacks in adults with episodic migraine. At 2 h postdose, pain-free rates ranged from 57.6% to 66.3%, pain relief response was 81.7% to 88.4%, and absence of the MBS ranged from 69.0% to 77.7% in the first 4 attacks treated with DFN-11. Freedom from migraine associated symptoms at 2 h ranged 78.1% to 86.5% for nausea, 72.3% to 77.5% for photophobia, and 73.6% to 77.5% for phonophobia. DFN-11 had a good tolerability profile, with a predictable but low incidence of TEAEs (ie, injection site reactions) that were mostly mild, and a very low incidence of triptan sensations (all considered noncardiac). These findings show that DFN-11 was consistently effective, tolerable, and safe in the acute treatment of multiple migraine attacks over an 8-week period.

With the caveat that comparing efficacy results from studies with different study populations and methodologies can be misleading, the magnitude of multiple-attack response to DFN-11 appears to be roughly comparable to the 6 mg SC dose of sumatriptan in published reports. For example, an 18-month open-label study of the 6 mg SC dose reported 2-h response rates of 67.0% for pain-free and approximately 72% for pain relief [16]. A trial evaluating sumatriptan SC 6 mg across 455 attacks in 100 consecutive patients found that 84% of subjects had pain relief at 2 h postdose [17]. In the current study of the effects of DFN-11 across 4 attacks, the range of 2-h pain-free rates was narrow and slightly lower (approximately 58–66%), but the range of pain relief responses was considerably higher (approximately 82–88%).

The results of this study confirm and extend the known safety profile of DFN-11. As expected, the overall rate of AEs was low, and only 1 subject experienced severe TEAEs. The most frequently reported TEAEs overall (injection site swelling and pain), as well as those associated with triptans (chest discomfort), decreased in incidence across the 4 treated attacks. Slightly more than one quarter (27.4%) of subjects treated with DFN-11 had at least 1 injection site reaction, which is less than half the rate reported with a 6-mg dose autoinjector (59%) [12] and about one third the incidence in a placebo-controlled study (79%) pooling data from 4 attacks [18]. The rate of injection site TEAEs may be related to the DFN-11 lower dose of active drug, dilute solution, and thin needle — the last of which has been shown to reduce pain and increase patient adherence [19]. Chest discomfort affected only 2.7% of subjects treated with DFN-11, and 5 of the 6 affected subjects reported only mild symptoms. Accounting for differences that might be expected due to the lower dose of sumatriptan in DFN-11 compared with traditional SC sumatriptan (3 mg vs 6 mg), this still represents a reduction from rates observed in earlier multiple-attacks studies of the 6 mg SC dose of sumatriptan. For example, in a previous study over a median of 25 months, 41% of subjects treated with a 6 mg dose of SC sumatriptan experienced chest symptoms in all attacks, and 39% had them in some attacks, leading 10% of subjects to discontinue sumatriptan [20]. In the placebo-controlled study of 4 attacks [18], 15.6% of subjects treated with the 6 mg dose of SC sumatriptan reported chest symptoms, an 83% increase versus DFN-11.

Limitations of this study include the open-label design and a possible selection bias for responders during the open-label period. Also, the study did not assess consistency of response within each individual. Despite these, the size of the study and consistency of response across 4 attacks suggest that DFN-11 will provide predictable migraine relief in clinical practice. In addition, the low incidence of TEAEs that generally decreased across the 4 attacks may increase patient confidence in positive results, encourage patient adherence to professional recommendations (eg, treating at the first sign of migraine pain) and, ultimately, contribute to better efficacy outcomes in clinical practice.

## Conclusions

DFN-11 was consistently effective, tolerable, and safe in the acute treatment of multiple migraine attacks over an 8-week period. The responses to DFN-11 at 2 h postdose on migraine pain freedom, pain relief, and associated symptoms (including the most bothersome) endpoints were substantial, and their range across attacks was narrow. TEAEs were predictable and mostly mild, with a very low incidence of triptan sensations. These findings underscore the potential of DFN-11 as an effective and safe SC sumatriptan option for the acute treatment of migraine.

## Abbreviations

AE: Adverse event
ECG: Electrocardiogram;
eDiary: Electronic diary
ICHD: International Classification of Headache Disorders
LOCF: Last observation carried forward
MBS: Most bothersome symptom
MedDRA: Medical Dictionary for Regulatory Activities
SAE: Serious adverse event
SC: Subcutaneous
SD: Standard deviation
TEAE: Treatment-emergent adverse event
WHODDE: World Health Organization Drug Dictionary Enhanced
